# Structural and Functional Plasticity at the Axon Initial Segment

**DOI:** 10.3389/fncel.2016.00250

**Published:** 2016-10-25

**Authors:** Rei Yamada, Hiroshi Kuba

**Affiliations:** Department of Cell Physiology, Graduate School of Medicine, Nagoya UniversityNagoya, Japan

**Keywords:** axon initial segment, plasticity, excitability, action potential, ion channel

## Abstract

The axon initial segment (AIS) is positioned between the axonal and somato-dendritic compartments and plays a pivotal role in triggering action potentials (APs) and determining neuronal output. It is now widely accepted that structural properties of the AIS, such as length and/or location relative to the soma, change in an activity-dependent manner. This structural plasticity of the AIS is known to be crucial for homeostatic control of neuronal excitability. However, it is obvious that the impact of the AIS on neuronal excitability is critically dependent on the biophysical properties of the AIS, which are primarily determined by the composition and characteristics of ion channels in this domain. Moreover, these properties can be altered via phosphorylation and/or redistribution of the channels. Recently, studies in auditory neurons showed that alterations in the composition of voltage-gated K^+^ (Kv) channels at the AIS coincide with elongation of the AIS, thereby enhancing the neuronal excitability, suggesting that the interaction between structural and functional plasticities of the AIS is important in the control of neuronal excitability. In this review, we will summarize the current knowledge regarding structural and functional alterations of the AIS and discuss how they interact and contribute to regulating the neuronal output.

## Introduction

The axon initial segment (AIS) is an excitable neuronal domain that separates the axonal and somatodendritic compartments and is involved in the initiation of action potentials (APs). The biophysical and structural characteristics of the AIS are considered ideal for AP initiation. In particular, the AIS has the lowest threshold for APs within neurons, due to the high density of voltage-gated Na^+^ (Nav) channels (for review, Debanne et al., 2011), which is accomplished through an interaction with a molecular complex composed of membrane scaffolds, cell adhesion molecules and cytoskeletal proteins (for review, Ogawa and Rasband, 2008). In addition, the AIS is located proximally in the axon; the isolation from the soma makes the domain electrically compact, while the proximity to the soma maximizes the charge reaching the domain from the soma, thereby increasing its excitability and making this domain further preferable for AP initiation (for review, Kole and Stuart, 2012). Importantly, these biophysical and structural characteristics of the AIS vary among individual neurons (for review, Kuba, 2012). Moreover, they even change in a manner dependent on neural activity, indicating that the AIS is a site of plasticity and contributes to the fine regulation of neuronal output (for review, Grubb et al., 2011). Furthermore, recent studies have revealed that these biophysical and structural changes can interact with each other, emphasizing the necessity of understanding the interactions to interpret their effects on the neuronal output.

## Biophysical Effects on Excitability

Our understanding of the mechanisms by which the AIS regulates neuronal activity was advanced greatly by various findings regarding the subtypes, distributions and roles of ion channels at the AIS.

Three types of Nav channels are found at the AIS. Among them, Nav1.6 is the most common subtype (Jenkins and Bennett, 2001; Lorincz and Nusser, 2008) and has the lowest activation threshold (Colbert and Pan, 2002; Rush et al., 2005), thus being the primary contributor to AP initiation in most neurons. Notably, Nav1.6 is located distally within the AIS (Van Wart et al., 2007; Hu et al., 2009), and this segregation from the soma also makes it suitable for AP initiation (Palmer and Stuart, 2006; Kole et al., 2008; Hu et al., 2009; Baranauskas et al., 2013). On the other hand, Nav1.1 and Nav1.2 are expressed in a cell-type-specific manner and are located proximally within the AIS (Van Wart et al., 2007; Lorincz and Nusser, 2008; Hu et al., 2009). One possible role of these subtypes is to promote back-propagation of APs to dendrites (Hu et al., 2009).

Several types of voltage-gated K^+^ (Kv) channels are reported at the AIS (Pan et al., 2006; Lorincz and Nusser, 2008). Since these Kv channels are expressed at different levels and in different combinations among neurons, they play a major role in determining the firing behavior of individual neurons (Johnston et al., 2010). In general, Kv1 (Kv1.1 and 1.2) and Kv7 (Kv7.2 and 7.3) channels have low activation thresholds and suppress AP generation by counteracting Nav channels either actively or passively as a shunt (Dodson et al., 2002; Goldberg et al., 2008; Shah et al., 2008; for review, Clark et al., 2009). In addition, Kv1 channels are known to be critical for shortening APs (Kole et al., 2007; Shu et al., 2007), and Kv7 channels contribute to maintaining availability of Nav channels by setting the resting potential (Battefeld et al., 2014). On the other hand, Kv2 (Kv2.1 and Kv2.2) channels have high activation thresholds. Therefore, Kv2 channels are preferentially activated by APs and promote repetitive firing by accelerating the repolarization of APs (Johnston et al., 2008).

Voltage-gated Ca^2+^ (Cav) channels, which were recently identified at the AIS, contribute to shaping the firing behavior of neurons in various ways. Cav2.3 and Cav3 have relatively low activation thresholds and promote AP generation by augmenting after-depolarization (Bender and Trussell, 2009), whereas Cav2.1 and Cav2.2 have high activation thresholds and suppress AP generation and facilitate AP repolarization by increasing shunting conductance via activation of Ca^2+^-activated K^+^ (BK) channels (Yu et al., 2010). Thus, multiple ion channels are expressed at the AIS, and their composition and distribution are strategically determined to shape the firing behavior of individual neurons.

## Structural Effects on Excitability

Structural characteristics of the AIS, such as length and distance from the soma, strongly affect the excitability and firing behavior of neurons (Kuba et al., 2006; Fried et al., 2009; Kuba and Ohmori, 2009; Kress et al., 2010). Effects of the AIS structure on neuronal excitability have been examined extensively in the nucleus laminaris, which is the third-order nucleus in the avian auditory pathway that is involved in sound localization (Kuba et al., 2006; for review Adachi et al., 2015). In this nucleus, the length of the AIS and its distance from the soma vary depending on the tuning frequency of the neurons, such that the AIS is shorter and more distal from the soma in neurons with higher tuning frequencies. This negative correlation between AIS length and distance from the soma is considered optimal for maximizing the excitability of the neurons.

The location of the AIS is related to the extent of its isolation from the soma, and greater distance increases the isolation. This isolation affects the excitability in two ways. First, it reduces the effects of the conductive and capacitive loads of somato-dendritic compartments on the AIS, thereby increasing the excitability. Second, it increases the dissipation of charges along the axon during propagation from the soma, thereby decreasing the excitability. Accordingly, the highest excitability of the AIS occurs at a certain distance from the soma, but a further increase in the distance reduces excitability because more charge is required to overcome the charge dissipation and generate APs (threshold current).

The surface area of the AIS is associated with the number of ion channels in this domain. This implies that elongation of the AIS increases both Na^+^ and K^+^ conductances in the AIS; the latter acts as a shunt around the resting potential (see above). In this situation, a longer AIS shows higher excitability, particularly when the AIS is located near the soma. This occurs because the increase in Na^+^ conductance at the AIS helps the domain to overcome the effects of somato-dendritic loads, while the short distance enables the AIS to overcome the large shunting conductance in the domain. However, when the AIS is located distally from the soma, the long AIS becomes less excitable because the charge dissipation increases with the distance, making it difficult to depolarize the AIS above the AP threshold in the presence of the shunting conductance. On the other hand, a shorter AIS becomes more excitable at a distal location because it has a relatively small shunting conductance, and the effects of somato-dendritic loads are milder in this location. Thus, a negative correlation between the AIS length and distance from the soma can maximize excitability, and this relationship is critically influenced by the conductances at both the AIS and the somato-dendritic compartments. The importance of somato-dendritic compartments to this relationship is also reported in models with realistic neuronal morphologies (Gulledge and Bravo, 2016).

## Biophysical Modulation of the AIS

Biophysical and structural characteristics of the AIS can change over different time scales, which endows neurons with efficient ways of adjusting their output. In many cases, biophysical changes are mediated through the modulation of ion channels via synaptic potentials and/or the activation of metabotropic receptors in the AIS and can proceed within milliseconds or seconds.

## Modulation Via Ionotropic Receptors

Glutamatergic excitatory synapses are not formed at the AIS. However, barrages of excitatory inputs to the somato-dendritic compartments temporally summate and cause a prolonged depolarization at the AIS. This alters the balance between Na^+^ and K^+^ conductances and affects the output of neurons. One prominent example is the inactivation of Kv1 channels during the depolarization, which broadens APs at the AIS and strengthens the synaptic output from cortical pyramidal neurons (Kole et al., 2007; Shu et al., 2007). Nav channels are also subjected to inactivation during the prolonged depolarization. This inactivation decreases with increasing distance from the soma, due to an electrotonic decrement of the depolarization. Accordingly, neurons overcome the effects of this inactivation and maintain their excitability by transient expansion of AP initiation areas at the AIS (hippocampus, Scott et al., 2014), or by localizing the AIS at a distal location (nucleus laminaris, see above, Kuba et al., 2006).

In cortical and hippocampal excitatory neurons, the AIS is innervated by GABAergic fast-spiking interneurons, and AP generation is directly modulated at the AIS (for review, Howard et al., 2005). This axo-axonic GABAergic input depolarizes the membrane (Szabadics et al., 2006; Woodruff et al., 2006) because potassium-chloride co-transporters, KCC2, which are the key molecules to extrude intracellular Cl^−^, are very few at the AIS (Szabadics et al., 2006; Khirug et al., 2008). Although it is still debated, this GABAergic input has been suggested to decrease the output of neurons via an increase in the shunting conductance at the AIS, particularly during *in vivo*-like membrane potential depolarization (Woodruff et al., 2011).

## Modulation Via Metabotropic Receptors

Several metabotropic modulations are reported at the AIS. Modulation of Nav channels is mediated via 5HT_1A_ receptors (Cotel et al., 2013). Notably, however, the effects of this serotonergic modulation differ among neurons. In cortical pyramidal neurons, activation of 5HT_1A_ receptors decreases Na^+^ conductance at the AIS via a positive shift in the activation curve of Nav channels (Yin et al., 2015). As this effect is specific to Nav1.2, this modulation suppresses back-propagation of APs with minimal effects on forward propagation in the axons. On the other hand, in auditory neurons of the medial superior olivary nucleus, activation of 5HT_1A_ receptors increases Na^+^ conductance at the AIS and enhances AP generation (Ko et al., 2016). This occurs because activation of these receptors inhibits hyperpolarization- and cyclic-nucleotide-gated (HCN) channels, thereby hyperpolarizing the membrane at the AIS and decreasing the inactivation of Nav channels.

Cav channels are also the targets of modulation. In inhibitory interneurons of the dorsal cochlear nucleus, Cav3.2 is coupled with dopaminergic D3 receptors at the AIS and is inhibited via the activation of protein kinase C in a β-arrestin-dependent manner (Bender et al., 2010; Yang et al., 2016). Consequently, dopamine suppresses burst firing by eliminating after-depolarization and reduces neuronal output. In hippocampal granule cells, on the other hand, Cav3.2 is activated by muscarinic M1 receptors at the AIS and elevates [Ca^2+^]_i_ in this domain (Martinello et al., 2015). This elevation of [Ca^2+^]_i_ causes a negative shift in the activation curve of Kv7 channels, thereby reducing the shunting conductance and augmenting AP generation. Thus, ion channels at the AIS are modulated in a cell-type-specific manner, which enables the appropriate adjustment of output in individual neurons.

## Structural Plasticity of the AIS

Structural characteristics of the AIS change depending on neuronal activity, which contributes to homeostatic control of excitability. This is rather slow in time course, and it requires hours or days to proceed. Two types of structural plasticity of the AIS are reported.

## Change in Location

Changes in AIS location were first observed in dissociated hippocampal culture (Grubb and Burrone, 2010; Evans et al., 2013). With chronic depolarization (2 days) via photo-stimulation or high [K^+^]_o_ media, all types of excitatory neurons in the culture move the entire AIS distally, resulting in a decrease in their excitability. Importantly, in dissociated culture of the olfactory bulb, chronic depolarization causes reciprocal movements of the AIS in excitatory and inhibitory neurons; the AIS moves proximally in inhibitory interneurons, whereas it moves distally in excitatory neurons (Chand et al., 2015), implying the cell-type-specific nature of the AIS plasticity. These reciprocal movements of the AIS may counterbalance the excess activity in the circuit. Indeed, changes in AIS locations are found in various disease models that are associated with hyperexcitability of neurons, such as epilepsy and demyelination (Harty et al., 2013; Hamada and Kole, 2015), supporting the idea that the AIS plasticity works as a negative-feedback mechanism to maintain homeostasis of the neural circuit.

## Change in Length

Changes in AIS length are also observed in pathological conditions. In the avian cochlear nucleus, in which neurons are innervated by the auditory nerve and send their projections to nucleus laminaris, the AIS elongates within several days after deprivation of auditory input (Kuba et al., 2010). This elongation adds Na^+^ conductance to the distal location and enhances the excitability, allowing the neurons to compensate for the loss of auditory nerve activity. Elongation also occurs in a mouse model of Angelman syndrome, which is a neurodevelopmental disorder associated with autism (Kaphzan et al., 2011), whereas shortening occurs after demyelination, stroke and traumatic brain injury (Baalman et al., 2013; Harty et al., 2013; Hamada and Kole, 2015). These alterations in AIS length would be critical in adjusting activity and maintaining homeostasis of neural circuits. In addition to the pathological conditions, length of the AIS changes in immature animals, particularly after the appearance of afferent inputs, implicating the involvement of AIS plasticity in the refinement of neural circuits during development (Cruz et al., 2009; Gutzmann et al., 2014; Kuba et al., 2014).

## Biophysical Interaction During Structural Plasticity

Recent studies have revealed that structural plasticity of the AIS coincides with changes in expression and/or modulation of ion channels at the domain. This is reasonable because the effects of AIS structure on excitability are tightly regulated by biophysical characteristics of the AIS.

**Figure 1:**
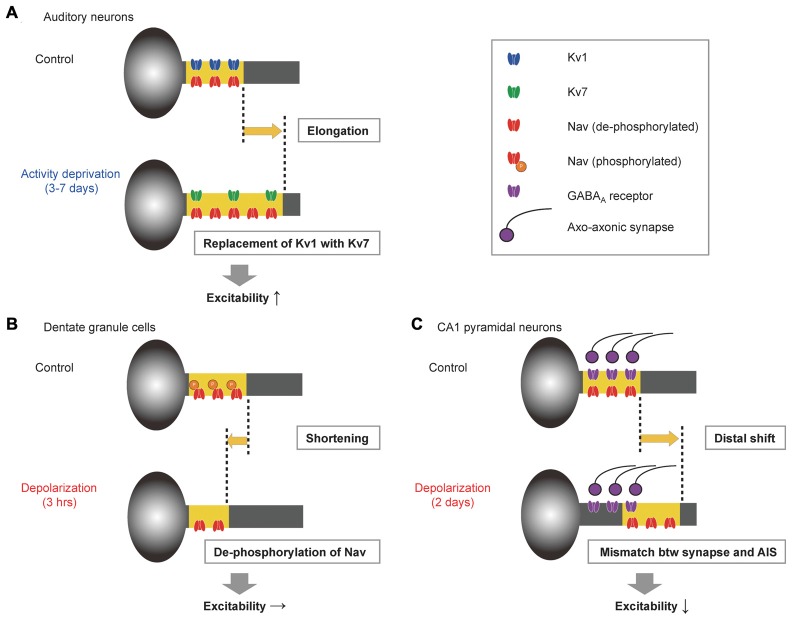
**Examples of structural and biophysical interaction during axon initial segment (AIS) plasticity. (A)** In neurons of the avian cochlear nucleus, elongation of the AIS is accompanied by replacement of Kv1 with Kv7 at the AIS, which augments the effects of elongation on excitability (Kuba et al., 2015). **(B)** In hippocampal dentate granule cells, depolarization for 3 h shortens the AIS but simultaneously de-phosphorylates voltage-gated Na^+^ (Nav) channels, which offsets the effects of shortening on excitability (Evans et al., 2015). **(C)** In hippocampal CA1 pyramidal neurons, depolarization for 2 days moves the AIS distally, but axo-axonic synapses remain at the original location, augmenting the suppressive effects of distal movement on excitability (Wefelmeyer et al., 2015).

In the avian cochlear nucleus, deprivation of auditory inputs elongates the AIS (see above). This elongation is accompanied by subtype-specific changes in expression of Kv channels: Kv1 decreases, whereas Kv7 increases at the AIS, resulting in complementary changes in their expression levels (Figure 1A; Kuba et al., 2015). Kv1 has rapid kinetics and is activated strongly with depolarization, while Kv7 has slow kinetics and behaves rather like passive conductance. Accordingly, these complementary changes in Kv channels reduce the shunting conductance during AP initiation, allowing the elongated AIS to compensate for the loss of auditory inputs more efficiently, with minimal effects on resting potential. This implies that these structural and biophysical changes in the AIS work synergistically and maintain the homeostasis of central auditory circuits after hearing loss.

In dentate granule cells of hippocampal cultures, on the other hand, structural and biophysical changes in the AIS are antagonistic (Figure 1B; Evans et al., 2015). Depolarizing the neurons for 3 h causes a shortening of the AIS, which is accompanied by de-phosphorylation of Nav channels in this domain; the shortening reduces excitability, whereas the de-phosphorylation of Nav channels increases excitability. Accordingly, these alterations counteract each other and maintain the excitability at a constant level. Nevertheless, the physiological role of this interaction remains elusive.

Notably, a longer depolarization (2 days) moves the AIS distally and suppresses excitability in the hippocampal neurons (see above; Evans et al., 2013). Although this movement is not accompanied by changes in the ion channel composition at the AIS (Grubb and Burrone, 2010), nor in the location of axo-axonic GABAergic synapses (Figure 1C; Wefelmeyer et al., 2015), it results in a spatial mismatch between the axo-axonic synapses and the AIS, augmenting the suppressive effects of the AIS movement. This augmentation occurs because the remaining synapses increase the shunting conductance between the soma and the AIS and reduce the charges reaching the AIS.

Structural plasticity of the AIS depends on changes in [Ca^2+^]_i_ via L-type Ca^2+^ channels (Grubb and Burrone, 2010), but their downstream signaling differs according to the types of plasticity. Key molecules are the calcium-dependent phosphatase calcineurin and cyclin-dependent kinase 5 (cdk5); calcineurin mediates the distal movement of the AIS in hippocampal neurons (Evans et al., 2013), whereas cdk5 mediates the proximal movement in olfactory bulb inhibitory interneurons (Chand et al., 2015). Although the precise mechanisms by which these molecules reorganize the AIS structure remain unknown, the mechanisms may involve post-translational modification of AIS proteins (Yoshimura and Rasband, 2014). Interestingly, it has been reported that calcineurin causes shortening of the AIS and simultaneously de-phosphorylates Nav channels in dentate granule cells (Evans et al., 2015). In addition, a blockade of cdk5 decreases the length of the AIS-like structure in mushroom body neurons in *Drosophila* (Trunova et al., 2011), whereas accumulation of Kv1 at the AIS requires phosphorylation via cdk2 and/or cdk5 (Vacher et al., 2011). These findings may indicate that structural and biophysical changes in the AIS share the same signaling molecules.

## Conclusion

Biophysical changes in the AIS are rapid, proceeding within milliseconds or seconds, and they can therefore contribute to modulating neuronal signal processing. On the other hand, structural changes are much slower, generally on the order of days, and would contribute to stability or refinement of neural circuits. Further, it is now evident that some structural changes can occur with a much faster time scale (within hours), allowing them to interact with other forms of plasticity, including long-term potentiation or depression, and shape neuronal signal processing. In addition, biophysical changes in the AIS occur simultaneously with structural changes and determine the effects of modulation. This indicates that the structural and functional plasticities of the AIS provide multiple and efficient methods of regulating neuronal excitability over various time scales and would enable the fine adjustment of neural activity during both physiological and pathological conditions.

However, many questions remain. Why do different types of AIS plasticity occur in individual cell types, e.g., resizing with redistribution of Kv channels in auditory neurons vs. relocation without redistribution of ion channels in hippocampal neurons? What is the mechanism for determining the types of plasticity implemented? How is the plasticity temporally and spatially regulated? How does it interact with other forms of plasticity? It is also important to see the impact of AIS plasticity on the function of neural circuits as well as its roles in the signal processing of individual neurons. As the AIS is the site of AP generation, and given that its abnormalities are associated with various neuropsychiatric disorders (Wimmer et al., 2010; Buffington and Rasband, 2011), exploring these issues will help us to understand the mechanisms by which activity is regulated in the brain during health and disease.

## Author Contributions

HK and RY wrote the manuscript.

## Conflict of Interest Statement

The authors declare that the research was conducted in the absence of any commercial or financial relationships that could be construed as a potential conflict of interest.
